# Hapten-labeled DNA probes can be stored and used in fluorescence *in situ* hybridization for decades

**DOI:** 10.3389/fgene.2025.1569308

**Published:** 2025-05-30

**Authors:** Thomas Liehr, Niklas Padutsch, Stefanie Kankel

**Affiliations:** Jena University Hospital, Institute of Human Genetics, Friedrich Schiller University, Jena, Germany

**Keywords:** fluorescence *in situ* hybridization, molecular cytogenetics, indirect labeling, direct labeling, commercial probes, homemade probes

## Abstract

In molecular cytogenetics, fluorescence *in situ* hybridization (FISH) is the main technique used. In both research and diagnostics, FISH depends on well-defined and mapped DNA probes that produce brilliant signals with minimal background, visible in metaphases and/or interphases. Such probes are either ready-to-use and commercially available or provided as unlabeled DNA. The latter can be obtained by flow sorting, microdissection, or by cloning DNA segments into appropriate bacterial vectors. Labeling can be done with either nonfluorescent or fluorescent haptens. According to international guidelines, such FISH probes must have a minimum shelf life, which is only between 2 and 3 years in human genetic diagnostics. The Molecular Cytogenetics Laboratory reporting here has been purchasing, producing, using, and storing FISH probes since the 1990s. For this study, the available stock of approximately 25,000 labeled probes was screened. A total of 581 FISH probes, labeled and approved 1–30 years before reuse, were selected for this study; of these, 75 were commercially available probes labeled 1–20 years ago. All of these probes, stored in the dark at −20°C, worked perfectly well in the FISH method. Although only slight to no differences in exposure times were observed over the years for self-labeled homemade probes, commercial probes labeled with SpectrumOrange had shorter exposure times and maintained them over the years. DNA probes labeled with SpectrumAqua/diethylaminocoumarin showed bright labeling for the first 3 years and then faded. Accordingly, it can be assumed that self-labeled homemade and commercial FISH probes can be stored stably in the dark and at −20°C for at least 30 years or longer. There is no need to test approved probes on a slide after the official expiry date. In practice, this suggests that a FISH probe tube that has been approved can be used in diagnostics until it is empty; there is no need to dispose of these expensive probes at any point due to their age.

## 1 Introduction

Molecular cytogenetics is a field that is mainly driven by the need to characterize chromosomal rearrangements as they occur in congenital and acquired human diseases [see (Weise and Liehr, 2017)]. Fluorescence *in situ* hybridization (FISH) was introduced into human cytogenetic diagnostics in the late 1980s (Pinkel et al., 1986); it was developed based on radioactive *in situ* hybridization, which had been available since 1969 (Gall and Pardue, 1969). FISH could only be developed after 1981, when non-radioactive probe labeling became available, such as biotin, which is detected by avidin coupled to a fluorochrome (Langer et al., 1981). Currently, in addition to the blue counterstain 4′,6-diamidino-2-phenylindole (DAPI), the following haptens and fluorochromes are used in routine applications of multicolor FISH (mFISH):

- biotin, detectable by avidin coupled with any type of fluorochrome mentioned below;
- digoxigenin, detectable by anti-digoxigenin coupled with any kind of fluorochrome mentioned below;
- SpectrumGreen;
- SpectrumOrange;
- SpectrumAqua/diethylaminocoumarin;
- Texas Red;
- Cyanine 5.

Furthermore, there are other less frequently used fluorochromes and haptens for mFISH applications, as described by Liehr (2025) and Sommerauer et al. (2017).

mFISH is used in diagnostics and research (Liehr 2025); FISH is needed to support diagnosis and monitor disease progression in leukemia, lymphoma, and solid tumors and characterize chromosome aberrations in congenital diseases (Weise and Liehr, 2017). DNA probes suitable for FISH applications include whole chromosome- and partial chromosome-targeted painting probes (whole chromosome-targeted painting probes (wcps) and partial chromosome-targeted painting probes (pcps)), centromere probes (CPs), and locus-specific probes (LSPs). wcps and pcps can be obtained through microdissection or flow sorting (Yang et al., 2017). CPs and LSPs are based on DNA sequences that are cloned into plasmids or bacterial artificial chromosomes (BACs) (Liehr 2017). For human FISH applications, DNA probes can be purchased from approximately a dozen suppliers worldwide. Most companies offer only labeled DNA probes; some, such as the BACPAC Resources Center (BPRC) (https://bacpacresources.org/), specifically offer LSPs as unlabeled DNA or as clones in *E. coli*.

In molecular cytogenetics, the use and application of FISH probes in research is relatively unrestricted. Researchers can label and use DNA probes at their discretion, provided they consider it appropriate for their specific environment. In diagnostics, the situation is completely different. Here, individuals from research backgrounds or from abroad often encounter unexpected obstacles in the use of molecular cytogenetics, such as the limited shelf life of FISH probes, which is only approximately 2 years. This is due to European legislation (EUR-Lex, 2025), which forces the industry even to set expiry dates for uncoated glass slides or salts that were previously stable underground for over 1,000,000 years, such as NaCl. However, any experienced clinical laboratory geneticist or technician who has been doing molecular cytogenetics for more than 10 years knows that FISH probes that have worked once and have been stored at −20°C in the dark practically never lose their ability to hybridize to a target DNA and practically always lead to evaluable results, regardless of their official expiration date.

Since the authors’ Molecular Cytogenetics Laboratory has been purchasing, manufacturing, using, and storing FISH probes since the 1990s, a study was conducted to scientifically prove the accuracy of the experience-based assessment of thousands of molecular cytogenetics professionals worldwide that a properly stored labeled DNA probe will virtually never lose its experimental effectiveness.

## 2 Material and methods

The Molecular Cytogenetics Laboratory, Jena, Germany, has a stock of approximately 25,000 labeled probes, including wcps, pcps, CPs, and LSPs. FISH probes have been produced or purchased since the 1990s. In this study, the available stock was manually inspected, with a focus on older probes. Finally, 581 FISH probes (see Table 1; Supplementary Tables S1–S3) were identified as labeled and first approved for anticipated use from 1 to 30 years ago. All these probes were used successfully beyond the registered time periods, as documented in the internal laboratory quality management system, which is approved under ISO 15189. All probes were stored at −20°C in the dark between their first and second use. A total of 506 probes were self-labeled homemade probes (between 1 and 20 years old), and 75 were commercially available probes (between 1 and 20 years old). For the sake of simplicity, all probes that are less than 12 months old have been grouped into the category of 1-year-old probes. The homemade probes were labeled using deoxyuridine triphosphates (dUTPs) tagged with the corresponding haptens (Liehr 2017). Of the probes, 40/506 and 0/75 were microdissection (midi)-derived wcps or pcps; 1/506 and 33/75 probes were CPs; and 42/75 and 465/506 were LSPs. The haptens were unevenly distributed across the entire probe collection due to factors such as the need for additional labeling beyond the most commonly used haptens—biotin (199/581), digoxigenin (168/581), SpectrumGreen (61/581), and SpectrumOrange (102/581). Texas Red (27/581) was less frequently used as it may also produce signals in the SpectrumOrange filter; SpectrumAqua/diethylaminocoumarin (29/581) has only been available for ∼10 years and is thus less frequently stocked. Similarly, the uneven distribution of commercial providers of probes reflects the differing needs to test different target regions, rather than differences in the quality of the DNA probes or a preference for a provider; among the probes included, 24/75 were from Abbott/Vysis (A/V, Wiesbaden, Germany), 14/75 from Cytocell/Sysmex (C, Norderstedt, Germany), 10/75 from Kreatech/Leica (K, Wetzlar, Germany), 1/75 from MetaSystems (M, Altlussheim, Germany), and 7/75 from ZytoVision (Z, Bremerhaven, Germany). All 581 probes were successfully used in routine diagnostics and produced bright, analyzable signals, which were documented using a standard image acquisition system (ISIS, MetaSystems, Altlussheim, Germany). Exposure times for 68 probes were determined for this study using the “Histogram” function of ISIS.

**TABLE 1:** Self-labeled homemade and commercial probes included in this study categorized based on age and labeling.

	Years in between (bio)	Years in between (dig)	Years in between (SG)	Years in between (SO)	Years in between (TR)	Years in between (SA)
Self-labeled homemade probe						
Range [y]	1–30	1–29	1–13	1–15	3–18	1–9
Number of cases	200	167	27	79	14	19
Commercial probe						
Range [y]	n.a.	n.a.	1–20	1–19	4–15	1–8
Number of cases	n.a.	n.a.	32	21	12	10

Abbreviations: bio, biotin; dig, digoxigenin; SG, SpectrumGreen; SO, SpectrumOrange; TR, Texas Red; SA, SpectrumAqua.

## 3 Results

**FIGURE 1:**
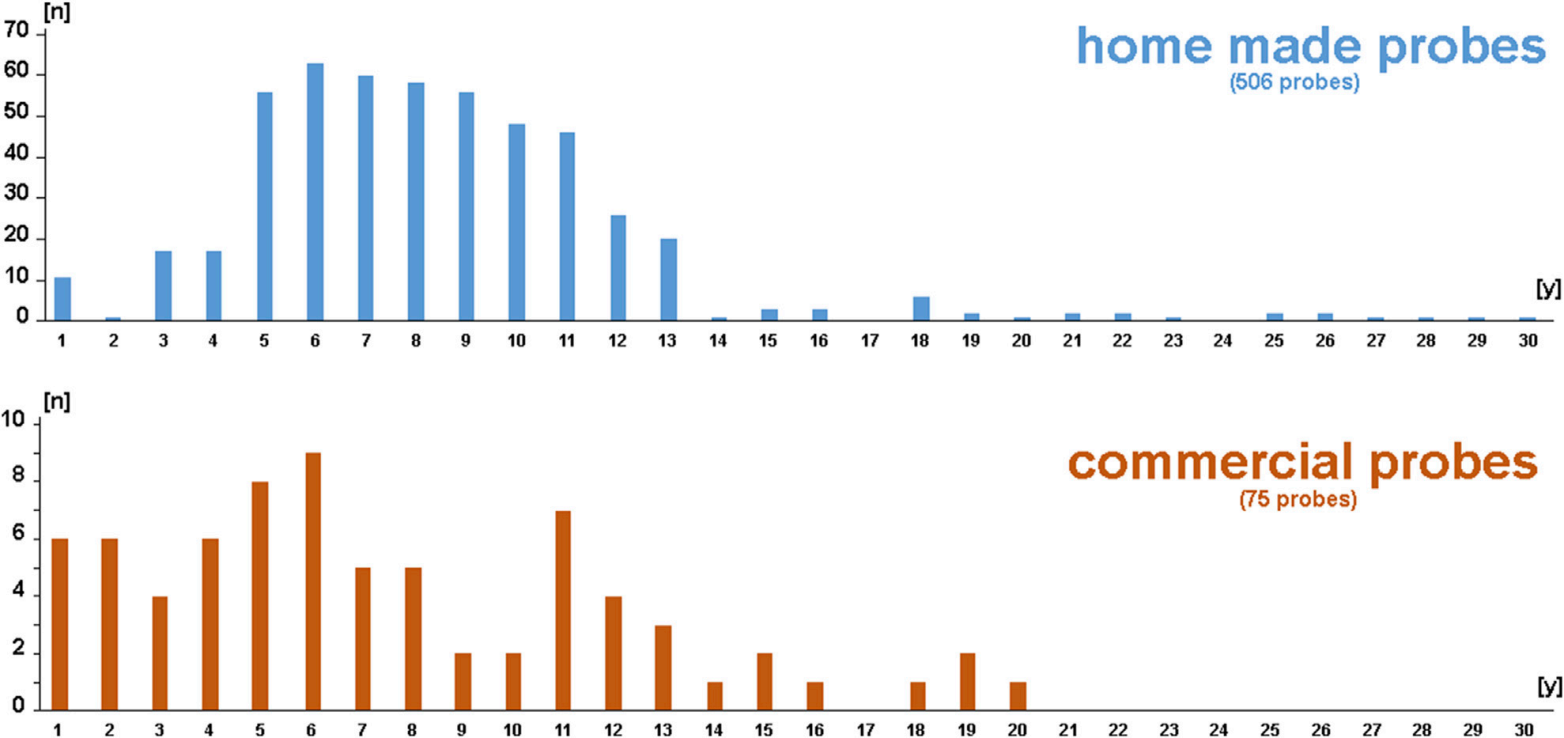
Schematic depiction of the 581 FISH probes included in this study. Among them, 506 were self-labeled homemade and 75 were commercial probes. The number of probes is sorted based on age (between 1 and 30 years).

Figure 1 shows a summary of the 581 FISH probes included, divided into 506 self-labeled homemade and 75 commercial probes. The time between labeling/first test and second successful use is given in years (1–30 years). None of the 581 probes that have proven functional in the past have failed during the second- use, regardless of the time elapsed.

**FIGURE 2:**
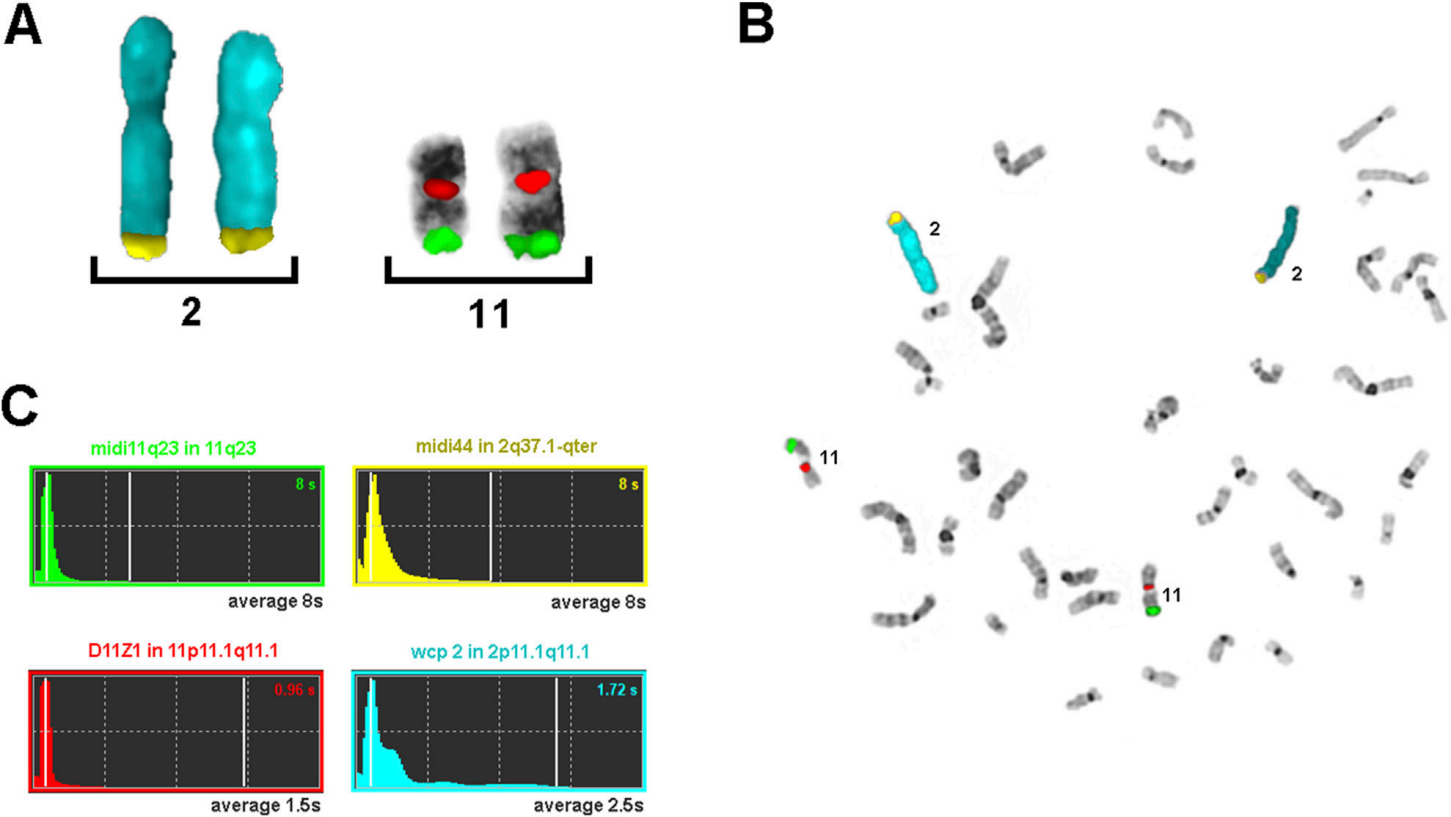
Results of a typical FISH experiment with probes of different ages. The wcp2 probe (blue) was 2.5 years old, the centromere 11 probe (orange) was 9 years old, and the pcp probes midi44 (biotin) and midi 11q23 (digoxigenin) were 25 and 30 years old, respectively. **(A)** Partial metaphase containing only the stained chromosomes and **(B)** a complete metaphase. **(C)** The exposure times used by the ISIS system (MetaSystems) are shown for **(B)**, together with the names and position of the four probes used; the average exposure times for 10 metaphases are represented in gray.

Figure 2 demonstrates the results of a typical FISH experiment using a mixture of probes of different ages: a 2.5-year-old wcp2 probe labeled with SpectrumAqua/diethylaminocoumarin; a nine-year-old commercial probe for centromere 11 labeled with SpectrumOrange; and two microdissection-derived pcps, midi44 and midi 11q23, which are 25 and 30 years old, respectively, labeled with biotin and digoxigenin. Regardless of the different exposure times determined by the ISIS system, all probes provide brilliant, easily interpretable FISH results.

**FIGURE 3:**
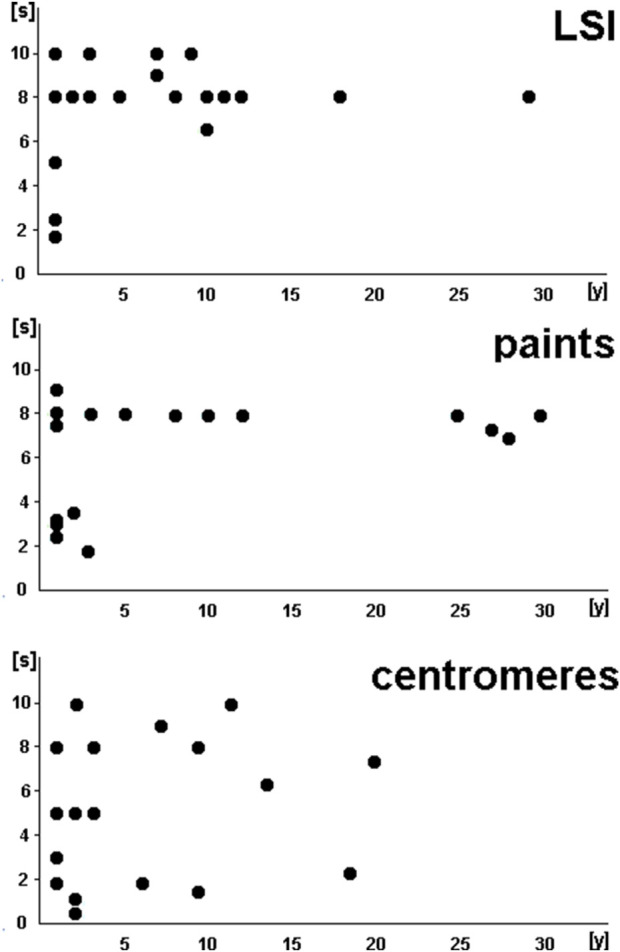
Exposure times of the 68 selected probes (see Supplementary Tables S1–S3) summarized based on the probe type. LSPs, locus-specific probes; paints, probes whose names start with wcp, pcp, or midi; centromeres, satellite DNA probes in centromeric and other regions of the human genome.

**FIGURE 4:**
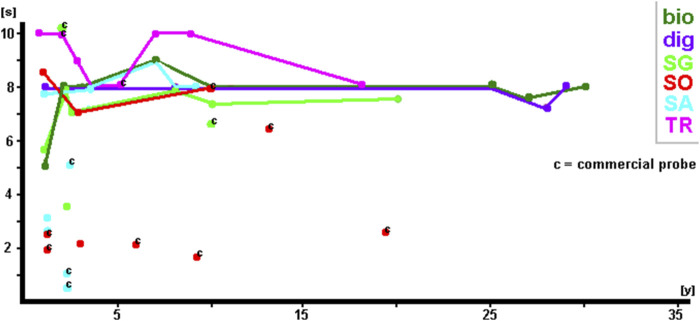
Exposure times of the 68 selected probes (see Supplementary Tables S1–S3) summarized by probe labeling.

Figures 3, 4 summarize the exposure times of the 68 selected probes (see Supplementary Tables S1–S3), categorized based on probe type (Figure 3) and labeling (Figure 4).

LSI and paint probes showed comparable patterns in exposure times: by 3 years of age at the latest, these probes required exposure times of 8–10 s; before that, approximately half of the included probes showed clearly stronger signals, requiring shorter exposure times of only ∼2–5 s. However, CPs showed variability in exposure times that was independent of age, ranging from less than 1 s to as much as 10 s.

Figure 4 shows that irrespective of labeling, exposure times were, in general, the same at ∼8–10 s. It must be noted that haptens such as biotin and digoxigenin were detected using corresponding fluorochrome-tagged antibodies. As no difference in signal intensity was detected, regardless of whether SpectrumGreen, SpectrumOrange, Texas Red, or Cyanine5 was used, only the primary labeling of non-fluorescent haptens is included in the results shown in this study (including Table 1; Supplementary Tables S1–S3). In Figure 4, the commercial probes are highlighted by a “c.” Even though there is one SpectrumGreen-labeled and one Texas Red-labeled probe with exposure times of 10 s 2 years after labeling, the majority of these probes have exposure times below 4 s. One SpectrumOrange-labeled probe even needed only 3 s exposure time at 15 years of age. In addition, it is noteworthy that self-labeled and commercial SpectrumAqua/diethylaminocoumarin-labeled probes produced extremely brilliant results during the first 3 years and returned to average exposure times of ∼8 s thereafter.

Overall, all 581 probes stored in the dark at −20°C performed perfectly well in the FISH method, even after 20 (commercial probes) or 30 years (homemade probes).

## 4 Discussion

Quality control is necessary as we also emphasized for FISH a few years ago (Liehr, 2010). However, some regulations are difficult to understand (such as setting expiration dates for uncoated glass slides or salts) or may even hinder high-quality diagnostics (Liehr, 2020). The latter refers to actual changes in the European Union’s “*In Vitro* Diagnostic Regulation (IVDR).” These raise the question, particularly in the field of molecular cytogenetic diagnostics, of whether it can still be offered to patients who urgently need their results. Unless FISH diagnostics for patients with unique chromosome aberrations is exempted from the IVDR as a specific form of “rare disease,” there is a risk of unnecessarily losing access to high-performance diagnostic tools (Liehr, 2020).

The actual study was conducted to support accreditation bodies in their review of FISH laboratories in human genetics and pathology. However, the annual checks by organizations in Germany through the German accreditation body DAkkS (Deutsche Akkreditierungsstelle) may even require that each expired FISH probe be tested on a normal control before another use in patients. If this test leads to good results in controls, the probe can be released for use for another 2 years. This must be repeated every 2 years and means that up to 50% of the commercial probes have to be used for such control experiments; for one commercial and one self-labeled probe, one has to pay 30–70 and 10–20 euros, respectively. The results of this study show that such tests are not necessary. At the very least, the use of “expired probes” on patient samples should be allowed and accepted as a basis for further use of the FISH probe in diagnostics. It is also clear that a demand, as made a few years ago by an inspector in Germany to a laboratory, to dispose of all (∼100) expired commercial FISH probes, is not reasonable and not justified by any scientific evidence.

In addition, the aspect that chromosomal aberrations are, in many cases, unique—or, at best, rare events must also be considered. This is best explained by an example: even though the laboratory Jena specializes in small supernumerary marker chromosomes (sSMCs), the necessary probe sets for characterization of an sSMC derived from chromosome 3 were applied between 2014 and 2019, only once per year, and since then, only in 2022 again. Had the available homemade five-color FISH probe set (subcenM-FISH) been considered expired after 3 years in 2022, as per standard guidelines, the costs per patient would be in the range of >700 €. This is as per single probe 10 tests need to be labelled; even when considering only 10 € per test, labelling of a new subcenM-FISH probe set for chromosome 3 would cost 500 € and would then be likely discarded unused. Additionally, one needs to use the cenM-FISH probe set (per test ∼200 €) to know which subcenM-FISH probe set is appropriate. The maximum reimbursement in Germany for FISH is 500 €; thus, this test could no longer be offered more cost-efficiently.

Nevertheless, it is recommended that all commercially available FISH probes be tested on a metaphase to verify their exact localization before diagnostic use. In 2021, the laboratory reporting had the following experience: the commercial probe BRCA1/17q, which was marketed by the supplier for interphase FISH on formalin-fixed paraffin-embedded (FFPE) sections of cancer patients, was ordered and tested as part of a research study exclusively on human cancer cell lines. In the metaphases examined, the commercial “BRCA1 probe” showed a very weak signal on chromosome 17q21.31, where it was expected to show up, and a very strong signal on 13q13—most likely it was mixed with the probe for BRCA2, which is also available from the same supplier and located in 13q13.1. In interphase, only the strong signal was visible—the signal on chromosome 13. The provider of the responsible company replied that the batch we had used had expired 15 months earlier, and therefore, they would not take responsibility for these results and refused to exchange the tube or take other actions. As shown in the actual work, the expired shelf lifetime cannot be the reason for this observed misallocation. Therefore, we concluded that all FISH probes used exclusively for interphase FISH need to be checked in metaphases before use in interphase research and diagnostics.

## 5 Conclusion

According to our literature search, there is not yet a single paper on the shelf life of FISH probes. It appears that freshly labeled FISH probes tend to yield more intense results than probes that are more than 3 years old. However, other factors, such as the age of the prepared chromosomes, the protein load of the chromosome preparations, and/or as-yet-unknown factors affecting chromosome quality, have a greater impact on the final FISH result than the age of the FISH probes. Based on the data from this publication, there is no need to test once-approved FISH probes after the official expiry date. In practice, this suggests that a once-approved FISH probe tube can even be used in diagnostics until it is empty, as long as storage conditions in the dark at −20°C are maintained. This is anyway standard for all FISH probes in all fields used. There is no need to dispose of the expensive probes at any time due to their age. As far as quality management provides for a re-examination of an officially expired probe, its functionality can also be easily demonstrated on patient samples. This is especially important considering that FISH probes are applied to investigate the causes of rare diseases.

## Data Availability

The original contributions presented in the study are included in the article/Supplementary Material; further inquiries can be directed to the corresponding author.

## Abbreviations

A/V, Abbott/Vysis; BPRC, BACPAC Resources Center; BACs, bacterial artificial chromosomes; bio, biotin; C, Cytocell/Sysmex; CP, centromere probe; DAkkS, Deutsche Akkreditierungsstelle; DAPI, 4′,6-diamidino-2-phenylindole; dig, digoxigenin; dUTP, deoxyuridine triphosphate; FISH, fluorescence *in situ* hybridization; FFPE, formalin-fixed paraffin-embedded; IVDR, *In Vitro* Diagnostic Regulation; K, Kreatech/Leica; LSPs, locus-specific probes; M, MetaSystems; mFISH, multicolor FISH; SA, SpectrumAqua; SG, SpectrumGreen; SO, SpectrumOrange; TR, Texas Red; pcps, partial chromosome-targeted painting probes; wcps, whole chromosome-targeted painting probes; Z, ZytoVision.
